# Low-Pathogenic Avian Influenza Viruses in Wild House Mice

**DOI:** 10.1371/journal.pone.0039206

**Published:** 2012-06-15

**Authors:** Susan A. Shriner, Kaci K. VanDalen, Nicole L. Mooers, Jeremy W. Ellis, Heather J. Sullivan, J. Jeffrey Root, Angela M. Pelzel, Alan B. Franklin

**Affiliations:** National Wildlife Research Center, United States Department of Agriculture (USDA) Animal and Plant Health Inspection Service, Fort Collins, Colorado, United States of America; Centers for Disease Control and Prevention, United States of America

## Abstract

**Background:**

Avian influenza viruses are known to productively infect a number of mammal species, several of which are commonly found on or near poultry and gamebird farms. While control of rodent species is often used to limit avian influenza virus transmission within and among outbreak sites, few studies have investigated the potential role of these species in outbreak dynamics.

**Methodology/Principal Findings:**

We trapped and sampled synanthropic mammals on a gamebird farm in Idaho, USA that had recently experienced a low pathogenic avian influenza outbreak. Six of six house mice (*Mus musculus*) caught on the outbreak farm were presumptively positive for antibodies to type A influenza. Consequently, we experimentally infected groups of naïve wild-caught house mice with five different low pathogenic avian influenza viruses that included three viruses derived from wild birds and two viruses derived from chickens. Virus replication was efficient in house mice inoculated with viruses derived from wild birds and more moderate for chicken-derived viruses. Mean titers (EID_50_ equivalents/mL) across all lung samples from seven days of sampling (three mice/day) ranged from 10^3.89^ (H3N6) to 10^5.06^ (H4N6) for the wild bird viruses and 10^2.08^ (H6N2) to 10^2.85^ (H4N8) for the chicken-derived viruses. Interestingly, multiple regression models indicated differential replication between sexes, with significantly (p<0.05) higher concentrations of avian influenza RNA found in females compared with males.

**Conclusions/Significance:**

Avian influenza viruses replicated efficiently in wild-caught house mice without adaptation, indicating mice may be a risk pathway for movement of avian influenza viruses on poultry and gamebird farms. Differential virus replication between males and females warrants further investigation to determine the generality of this result in avian influenza disease dynamics.

## Introduction

The emergence of highly pathogenic Asian strain H5N1 avian influenza virus has led to increased scrutiny of avian influenza viruses and a better understanding of the frequency with which avian influenza viruses spill over into mammalian populations [1], [2]. One of the earliest demonstrations of replication of avian influenza viruses in mammals was an experimental infection study with ferrets that showed ferrets could productively replicate non-adapted avian influenza viruses [3]. Later, the discovery of a natural infection of seals with an avian-like virus prompted an investigation of the general susceptibility of mammals to avian influenza viruses [4]. This study showed avian influenza viruses could replicate efficiently in pigs, ferrets, and cats. Since then, evidence of natural infection with avian influenza viruses has been found for a number of mammal species, including harbor seals [5], [6], whales [7], mink [8], [9], stone martens [10], raccoons [11], large wild cats [12], [13], domestic cats [14], [15], [16], [17], [18], [19], civets [20], domestic dogs [18], [21], pigs [22], [23], donkeys [24], and humans [25]. Experimental infection studies show an even broader range of mammalian species is susceptible to avian influenza virus infection. Laboratory mice (*Mus musculus*) [26], [27], [28], [29], laboratory rats (*Rattus norwegicus*) [30], thirteen-lined ground squirrels [31], ferrets [32], striped skunks [31], rabbits [33], red foxes [34], macaques [35], and cattle [36] have all been shown to replicate avian influenza viruses.

These findings have led to a growing recognition that wild mammals may contribute to the spread of avian influenza viruses. In particular, mammals associated with agricultural operations may represent a risk path for virus transmission within and among farms [1], [2], [37], [38], [39]. Some of the most prevalent mammal species on farms are nuisance species such as mice and rats. While no studies have shown a link between rodents and avian influenza virus transmission, rodent control is a recommended biosecurity measure for limiting the spread of avian influenza viruses on farms. Despite this concern that rodents pose a transmission risk, scant attention has been focused on elucidating the role rodents may play in outbreak dynamics on poultry and gamebird farms.

Many experimental infection studies have confirmed the ability of laboratory mice to productively replicate both poultry- and wild bird-derived avian influenza viruses [26], [27], [28], [29], [40], [41]. However, the primary goal of these studies has been to use mice as a surrogate for studying human disease and objectives have generally centered on demonstrating the utility of mice as an animal model for studying pathogenesis of different avian influenza subtypes, evaluating antiviral drugs, or screening various avian influenza strains for vaccine development. As such, experimental control was of prime importance in the design of these studies and all experimentation relied exclusively on the use of female laboratory BALB/c mice. Consequently, the results from these studies provide limited inference to viral replication in wild house mouse (*Mus musculus*) populations, which are far more heterogeneous than BALB/c mice. Natural populations are comprised of individuals with a myriad of demographic and physiological characteristics that could affect viral replication rates such as sex, age, nutritional condition, reproductive class, and disease status [42], [43]. In this study, we evaluated the potential role of common wild mammals in avian influenza outbreaks on poultry facilities in two ways: 1) we surveyed synanthropic mammals at an avian influenza outbreak site, and 2) we experimentally infected wild-caught house mice with avian influenza viruses.

## Methods

### Ethics Statement

All experiments were approved by the Institutional Animal Care and Use Committee of the United States Department of Agriculture, Animal and Plant Health Inspection Service, Wildlife Services, National Wildlife Research Center (NWRC), Fort Collins, CO, USA. (Approval numbers NWRC 1512 and NWRC 1620).

### Low-pathogenic Avian Influenza Outbreak

In August 2008, low pathogenic avian influenza (LPAI) H5N8 virus was isolated from a flock of breeding and raised-for-release upland game birds maintained in a combination of indoor and outdoor pens in southwest Idaho. Portions of the flock of approximately 33,000 pheasant, mallard ducks, chukar partridge, quail, and pigeons were also concurrently infected with two other LPAI viruses, H4N7 and H11N7. The flock was quarantined by the Idaho State Department of Agriculture, an epidemiological investigation was conducted, and regulatory response activities were performed in accordance with Idaho's H5/H7 LPAI Initial Response and Containment Plan using both state (ISDA) and federal (USDA-APHIS-Veterinary Services) personnel.

The 27 domestic flocks located within 3 kilometers of the infected flock were quarantined and underwent two rounds of both serologic and antigen testing for avian influenza (AI) with all negative results. All premises identified during the epidemiological investigation as traces or dangerous contacts with the index flock were either tested AI negative or voluntarily depopulated. There was no spread of infection identified beyond the index flock. Results of the epidemiological investigation indicated the most likely source of AI infection on the index premises was wild migratory waterfowl interacting with the domestic ducks in the outdoor pens.

The infected flock was depopulated and facilities on the premises were thoroughly cleaned and disinfected. Sentinel birds were placed on the index premises and sampled for 21 days with negative results for AI. The index premises were then fully repopulated with birds and underwent enhanced active surveillance testing for several weeks after placement with all negative AI results. The index premises were released from quarantine in December 2008.

### Mammal Survey at the Outbreak Site

In response to the described OIE (World Organization for Animal Health) reported low-pathogenic avian influenza outbreak at a gamebird farm in Idaho in the fall of 2008, we conducted a small scale survey of wild mammals at the outbreak site. Sampling occurred approximately one month after the initial birds were submitted for testing. Mammals were trapped at various locations on the farm (outside of bird pens) using Sherman folding traps and Tomahawk live-capture traps over the course of two nights. Oral swabs were collected from captured mammals and stored in BA-1 viral transport media (M199-Hank's salts, 1% bovine serum albumin, 350 mg/l sodium bicarbonate, 2.5 mg/mL amphotericin B in 0.05 M Tris, 100 units/ml penicillin, 100 mg/mL streptomycin, pH 7.6). We also collected serum samples from each animal and all samples were shipped on ice overnight for serological testing. In addition to the mammal samples, we collected approximately 50 fresh fecal samples from mallards located on the farm and approximately 50 water samples from the mallard pens. These samples were collected in order to determine whether avian influenza virus was present on the gamebird farm at the time of mammal sampling. Serum samples were tested for antibodies to influenza A viruses by an indirect ELISA and oral swabs, fecal swabs, and water samples were tested for avian influenza viral RNA via real-time reverse-transcription polymerase chain reaction (RRT-PCR) as described by VanDalen et al. [44].

### Viruses

We used five avian influenza viruses derived from North American wild birds or poultry (Table 1) for the experimental infections. Two of the viruses (A/Wild bird/CA/187718-36/08 [H3N8] and A/Mallard/OR/A00047710/08 [H3N6]) were collected from wild birds as part of an U.S. national surveillance system for avian influenza initiated in 2006 [45]. A third virus (A/mallard/CO/P66F1-5/08 [H4N6]) was originally collected from a wild bird as part of the U.S. surveillance, but was then passaged through a mallard prior to virus isolation in hen eggs. These three viruses were selected because they are among the most commonly isolated subtypes from North American waterfowl [46]. The remaining two viruses (A/CK/CA/S0408793/04 [H6N2] and A/CK/AL/75 [H4N8]) were derived from poultry and were provided courtesy of the U.S. Department of Agriculture, Agricultural Research Service, Southeast Poultry Research Laboratory (SEPRL), Athens, GA USA. These two viruses were selected to represent known poultry outbreaks. The H6N2 virus was originally collected from a chicken from a live bird market in southern California in 2004 and was selected as a representative of H6N2 viruses circulating in commercial poultry from 2000–2005 [47]. The H4N8 virus is from a well-studied 1975 avian influenza outbreak on three poultry farms in Alabama [48], [49], [50]. Virus stocks were propagated in the allantoic cavity of 9–11 day old specific pathogen free embryonated hen eggs at 37°C. Allantoic fluid was harvested, pooled, aliquoted and stored at −80°C prior to titration. Virus titers were determined as 50% egg infective dose (EID_50_) in 9–11 day old specific pathogen free embryonated hen eggs or as 50% tissue culture infective does (TCID_50_) via tissue culture in Madin-Darby canine kidney (MDCK) cells [51]. Viral titers were calculated using the Reed and Muench method [52]. Prior to use, the wild bird viruses were limited to a single passage in hen eggs to limit potential genetic adaptation while the chicken viruses had been passaged multiple times.

**Table 1:** Avian influenza viruses used in this study.

Virus	Subtype	Species origin	Sample Origin
A/Mallard/OR/A00047710/06	H3N6	Mallard	Cloacal swab
A/Wild bird/CA/187718-36/08	H3N8	Wild bird	Pooled fecal swabs
A/Mallard/CO/P66F1-5/08	H4N6	Mallard	Fecal swab
A/CK/CA/S0408793/04	H4N8	Chicken	Cloacal swab
A/CK/AL/75	H6N2	Chicken	Cloacal swab

### Experimental Animals

Wild house mice (*Mus musculus*, hereafter mice, N = 125) were live-trapped in Northern Colorado, USA using Sherman folding traps baited with peanut-butter and grain. Traps were opened in the evenings prior to sunset and checked in the early morning. Mice were primarily caught on farms and dairies in Larimer and Weld Counties. Upon accession at the National Wildlife Research Center Animal Research Building, mice were weighed, sexed, and dusted for parasites. Initial weights ranged from 5–30 g (median = 16.5 g) upon entry and the group included 62 females and 63 males. Mice were quarantined for a minimum of two weeks prior to testing; mice weighing less than 12 g upon entry were held for at least four weeks such that all mice tested were considered to be adults. Mice were tested for antibodies to type A influenza by indirect ELISA using Imgenex IMR-274 recombinant influenza A protein (nuclear protein NP) and Immunogen Rabbit anti-Mouse IgG-HRP. A single mouse tested positive and was not used in the study. Mice were individually housed and only one avian influenza virus subtype was tested per animal room.

### Experimental Inoculation

Groups of approximately 24 mice were randomly assigned to each of the five LPAI subtype groups (H3N6, H3N8, H4N6, H4N8, H6N2) and five mice were assigned to a negative control group. Mice were lightly anesthetized with isoflurane gas and inoculated intranasally with 50 µL of 10^5^ EID_50_ or 10^5^ TCID_50_ of one of the five viruses diluted in uninfected allantoic fluid. Inoculation titers were confirmed via RRT-PCR post inoculation. Control mice were mock inoculated with 50 µL of uninfected allantoic fluid.

One cohort (generally three individuals) from each subtype treatment group was sampled daily through 7 days post inoculation (dpi). An oro-pharyngeal swab, fecal sample (if available), and blood sample were collected from each mouse. Swabs and fecal samples were placed in BA-1 and stored at −80°C. Nasal turbinates, trachea, and lungs were harvested post-mortem and frozen to −80°C. In addition, a nasal wash was collected post-mortem by pipetting 25 µL BA-1 into the nasal cavity and then reclaiming the media, which was then stored at −80°C. A final cohort from each subtype treatment group was sampled on 21 dpi according to the same procedures. The same samples were collected from each of the control animals, which were sampled on 3 dpi (2 mice), 4 dpi (2 mice), and 14 dpi (1 mouse).

### Laboratory Testing

Oral and nasal swabs were tested for the presence of influenza A viral RNA by RRT-PCR. Viral RNA was extracted from samples using the MagMAX-96 AI/ND Viral RNA Isolation Kit (Ambion, Austin, TX). Nasal turbinates, trachea, and lung tissues required additional steps before the MagMAX-96 AI/ND Viral RNA Isolation Kit could be used. Approximately 50–100 mg of trachea and lung tissues were added to a microcentrifuge tube with 750 µL of TRIzol LS (Invitrogen Corp, Carlsbad, CA) and one copper (3–7 mm) bead. Nasal turbinates (≤10 mg) were combined with 250 µL viral transport media, 750 TRIzol LS, and one copper bead. Microcentrifuge tubes were loaded and balanced into Qiagen Mixer Mill 301 racks (QIAGEN Inc, Valencia, CA) and homogenized for two minutes at 20 Hz. Racks were rotated 180° and homogenized for another two minutes at 20 Hz. Tissue lysates were incubated at room temperature for five minutes and then 200 µL of chloroform (99.8+%; Thermo Fisher Scientific, Waltham, MA) was added to each microcentrifuge tube. Lysates were vortexed for 15 seconds and allowed to incubate at room temperature another 2–15 minutes to release RNA from tissues. Next, tissue lysates were centrifuged at 5,000× g for 10 minutes and the supernatant was transferred to a new RNase-free microcentrifuge tube. RNA was extracted from the supernatant using the MagMAX-96 AI/ND Viral RNA Isolation Kit following the manufacturer's instructions except for the inclusion of an additional wash step (Wash S) in between the manufacturer's recommended Wash 1 and Wash 2 steps. The additional wash solution was 2M NaCL and 2 mM EDTA (pH 4.0) with 100 µL added to each well of a 96-well extraction plate. RRT-PCR was performed as described in VanDalen et al. [44]. Calibrated controls with known viral titers (10^2^ EID_50_/mL–10^5^ EID_50_/mL) were also analyzed with RRT-PCR to construct 4-point standard curves. Sample viral RNA quantities were extrapolated from the standard curves and are presented as PCR EID_50_ equivalents/mL.

### Statistical Analysis

In order to compare viral replication across virus subtypes and in male and female mice, we developed multiple regression models that tested viral concentrations in lung tissues as a function of three variables: virus subtype (H3N6, H3N8, H4N6, H4N8, H6N2), sex (female or male), and day post inoculation (dpi). Viral concentrations were log transformed and standardized by the mean weight of the tissues tested. We tested all possible models including each of the main effect variables (subtype, sex, and dpi) and their interactions. We used corrected Akaike's Information Criterion (AICc) to compare models and identify the model that best explained the data [53]. Statistical analyses were conducted using R version 2.12.0 [54].

## Results

### Serological Survey at a Low-pathogenic Avian Influenza Outbreak Site

Fourteen mammals were captured at the outbreak site: 6 house mice (*Mus musculus*), 6 Norway rats (*Rattus norvegicus*), 1 harvest mouse (*Reithrodontomys megalotis*), and 1 deer mouse (*Peromyscus maniculatus*). All mammalian oral swabs, mallard fecal swabs, and mallard pen water samples were negative for avian influenza viral RNA. The six house mouse serum samples were positive for antibodies to type A influenza by indirect ELISA, but sera volumes were too low to conduct confirmatory hemagglutination inhibition tests. The remaining animals were all seronegative.

### Viral replication in Experimental Animals

All negative controls were negative for avian influenza viral RNA in lung, nasal turbinate, and trachea tissues. Negative controls were also negative for antibodies to avian influenza. No mortality, systematic weight loss, or changes in appearance (ruffled fur, hunched posture) were observed in inoculated mice.

#### Lungs

**Figure 1:**
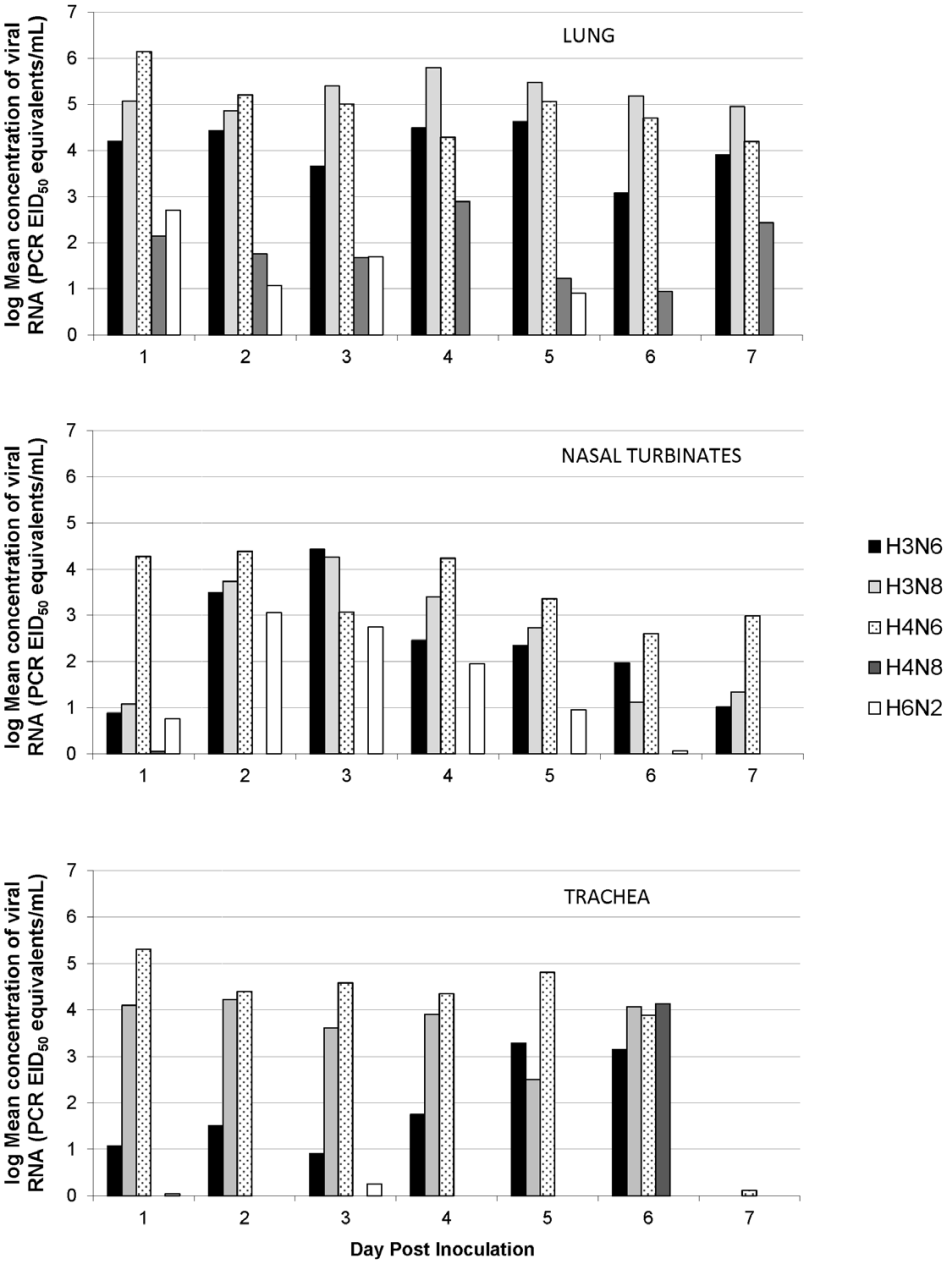
Comparison of avian influenza viral replication in three tissue types for five virus subtypes. Virus replication was more efficient in the lung tissues of mice inoculated with avian influenza virus subtypes derived from wild birds (H3N6, H3N8, and H4N6) compared with those derived from chickens (H4N8, H6N2). In general, three mice were tested for each tissue/day/virus.

Viral replication in lung tissues harvested from experimentally inoculated mice was efficient for the three wild bird viruses (H3N6, H3N8, and H4N6) and moderate (H4N8) or poor (H6N2) for the two poultry viruses (Figure 1). Concentrations of viral RNA were higher in lung tissues compared with nasal turbinates and trachea tissues for each of the subtypes other than the H6N2 virus, which replicated most efficiently in the nasal turbinates. Viral replication in lungs continued through 7 dpi for each of the viruses other than the H6N2 virus which showed peak replication on 1 dpi at 10^2.71^ EID_50_ equivalents/mL with only one individual between 4–7 dpi showing detectable virus (on 5 dpi). Peak concentrations occurred on 5 dpi for the H3N6 virus (10^4.62^ EID_50_ equivalents/mL), 4 dpi for the H3N8 virus (10^5.80^ EID_50_ equivalents/mL), 1 dpi for the H4N6 virus (10^6.15^ EID_50_ equivalents/mL), and 4 dpi for the H4N8 virus (10^2.90^ EID_50_ equivalents/mL). The percentage of inoculated mice with detectable levels of viral RNA in their lungs varied across subtypes from 35%–100% (Table 2).

**Table 2:** Frequency of avian influenza viral RNA detection (tissues) for the five viruses tested.

Lung	Subtype	DPI	1	2	3	4	5	6	7	Total
	H3N6		3	3	2	3	3	3	3	20 (95%)
	H3N8		3	3	3	3	3	3	3	21 (100%)
	H4N6		3	2	2	4*	2	3	2*	18 (86%)
	H4N8		2	3	2	2	2	1	2	14 (67%)
	H6N2		3	1	3	0	0	0	0*	7 (33%)

Nasal Turbinate	Subtype	DPI	1	2	3	4	5	6	7	Total
	H3N6		2	3	3	3	3	3	1	18 (86%)
	H3N8		3	3	3	3	3	2	1	18 (86%)
	H4N6		3	3	3	4*	3	3	2*	21 (100%)
	H4N8		1	1	0	1	0	0	0	3 (14%)
	H6N2		2	3	3	3	2	2	0*	15 (71%)

Trachea	Subtype	DPI	1	2	3	4	5	6	7	Total
	H3N6		2	3	2	2	2	1	0	12 (57%)
	H3N8		3	3	3	3	3	3	0	18 (86%)
	H4N6		3	3	3	4*	3	1	2*	19 (90%)
	H4N8		1	0	0	0	0	2	0	3 (14%)
	H6N2		2	0	3	0	0	0	0*	5 (25%)

* Three mice were tested for each tissue/day/virus subtype with the following exceptions: 4 dpi for H4N6 included 4 individuals, 7 dpi for H4N6 included 2 individuals, and 7 dpi for the H6N2 virus was 2 mice.

#### Nasal turbinates

The three wild bird viruses replicated efficiently in nasal turbinates while the H6N2 poultry virus showed moderate replication and the H4N8 was barely detectable (Figure 1). Replication continued through 7 dpi for the wild bird viruses and through 6 dpi for the H6N2 virus. Peak replication occurred on 3 dpi for the H3N6 virus (10^4.43^ EID_50_ equivalents/mL) and the H3N8 virus (10^4.26^ EID_50_ equivalents/mL) and on 2 dpi for the H4N6 virus (10^4.39^ EID_50_ equivalents/mL) and the H6N2 virus (10^3.06^ EID_50_ equivalents/mL). Viral RNA was detectable in 18 of 21 samples (86%) for the H3N6 and H3N8 viruses and in all of the H4N6 samples. The number of positive H6N2 nasal turbinate samples was 15/20 (75%), which was twice as many compared to lung tissues and five times the number of positive trachea samples, suggesting much stronger replication in the upper respiratory tract compared with the lower respiratory tract for the H6N2 virus.

#### Trachea

In almost all cases, viral RNA concentrations were lower in trachea tissues than in lung tissues, but concentrations showed a similar pattern of fairly efficient replication for the wild bird viruses and poor replication for the poultry viruses (Figure 1). However, compared with lung tissue replication, H3N6 concentrations were significantly lower than the other two wild bird viruses and replication of the two poultry viruses was almost non-existent (with the exception of a single individual that showed a concentration of 10^4.60^ EID_50_ equivalents/mL on 6 dpi for the H4N8 virus). In general, concentrations were higher in trachea tissues compared with nasal turbinates for the H3N8 and H4N6 viruses, but lower in trachea tissues compared with nasal turbinates for the other three viruses. Replication ceased by 7 dpi for all subtypes except for one H4N6 individual. Peak replication occurred on 5 dpi for the H3N6 virus (10^3.29^ EID_50_ equivalents/mL), 2 dpi for the H3N8 virus (10^4.23^ EID_50_ equivalents/mL), and 1 dpi for the H4N6 (10^5.31^ EID_50_ equivalents/mL). The percentage of inoculated mice with detectable levels of viral RNA found in tracheas varied from 14% for the H4N8 virus to 90% for the H4N6 virus (Table 2).

#### Nasal washes, oral swabs, and fecal samples

Results for nasal washes roughly corresponded to results for nasal turbinates with a similar number of samples exhibiting detectable viral RNA, but concentrations were generally 1–3 log_10_ lower (Table 3). All oral swabs were negative for mice inoculated with the H3N6, H4N8, and H6N2 viruses. For H3N8, 4 of 22 mice were positive (range = 10^0.56^–10^1.05^ EID_50_ equivalents/mL) and included 1 of 3 mice on 2 dpi, 2 of 3 mice on 4 dpi, and 1 of 3 on mice on 5 dpi. For H4N6, 3 of 22 mice were positive (range = 10^−0.66^–10^1.55^ EID_50_ equivalents/mL) and included 2 of 3 mice on 2 dpi and 1 of 3 mice on 3 dpi. All fecal samples were negative across the five subtypes tested.

**Table 3:** Mean avian influenza viral RNA concentrations in nasal washes.

DPI	1	2	3	4	5	6	7	21
H3N6	10^1.29^	10^1.94^	10^1.35^	10^2.37^	–	–	10^−1.10^	nd
	2/3	3/3	3/3	3/3	0/3	0/3	1/3	nd
H3N8	10^1.77^	10^2.91^	10^4.49^	10^3.04^	10^1.58^	10^2.48^	10^1.67^	–
	3/3	3/3	3/3	4/4	3/3	3/3	2/3	0/1
H4N6	10^1.57^	10^2.17^	10^3.96^	10^4.26^	10^3.67^	10^2.32^	10^3.07^	–
	2/2	3/3	3/3	3/3	2/2	3/3	2/2	0/3
H4N8	nd	–	10^−0.96^	–	10^2.09^	–	–	–
	–	0/3	1/3	0/3	1/3	0/3	0/3	0/3
H6N2	10^1.51^	10^0.73^	10^2.06^	10^1.15^	–	10^−0.59^	10^0.00^	–
	3/3	3/3	3/3	3/3	0/3	1/3	1/2	0/1

For each subtype tested, the first line reports the mean concentration of viral RNA (EID_50_ equivalents/mL) and the second line shows the number of positive swabs/number tested. – = not detected; nd = no data.

### Serology

None of the mice sampled on days 1–7 dpi were positive for influenza A antibodies by direct ELISA. For mice sampled on 21 dpi, differential immunogenicity was evident with positive results for only two of the five subtypes. Five of 15 mice were positive for influenza A antibodies: 3 of 3 mice inoculated with H4N6 were antibody positive and 2 of 3 mice inoculated with H4N8 virus were antibody positive.

### Statistical Analysis

Model selection results from the multiple regression models tested show the best model for viral RNA concentrations in lung tissues included virus subtype (p<0.005), sex (p<0.05), day post inoculation (p<0.05), and an interaction between sex and day post inoculation (p<0.05) (Table 4). For virus subtypes, the three wild bird viruses did not differ significantly from each other and the two poultry viruses did not differ significantly from each other, but the three wild bird viruses had significantly higher RNA concentrations in lungs compared with the two poultry viruses. Day post inoculation was also significant in the models but the relationship was primarily driven by the two poultry viruses, which showed near zero replication on days 6 and 7 dpi. On the other hand, replication rates for the wild bird viruses were relatively constant across days and were still relatively high on 7 dpi (Figure 1). When the poultry viruses and wild bird viruses were modeled independently, dpi was only selected as an important variable in the poultry virus models (p<0.05) and was not selected when only the first five days of sampling were included in the model.

**Table 4:** Model selection results for multiple regression models testing the relationship between viral RNA concentrations in lung tissues as a function of virus subtype, sex, and day post inoculation and interactions between the three variables.

Model	*K*	Adj. R^2^	AICc	ΔAICc	AICc weight
Subtype+Sex+DPI+Sex*DPI	9	0.62	374.3	0.0	0.46
Subtype+Sex+DPI+Subtype* Sex+Sex * DPI	13	0.63	377.1	2.8	0.11
Subtype+Sex+DPI+Subtype*DPI+Sex *DPI	13	0.63	377.1	2.8	0.11
Subtype+Sex+DPI	8	0.61	377.2	2.9	0.11
Subtype+DPI	7	0.60	378.6	4.3	0.05
Subtype+Sex	7	0.60	379.0	4.7	0.04
Subtype+Sex+DPI+Subtype*DPI	12	0.62	379.9	5.6	0.03

Only models with a ΔAICc<6 are shown. K is the number of parameters. Adj. R^2^ is the R^2^ value adjusted for the number of parameters in the model; it indicates the amount of variation explained in the model. AICc is Akaike's information criterion adjusted for small sample size. ΔAICc values indicate the difference between a given model and the best model. The AIC weight shows the relative support for each model.

**Figure 2:**
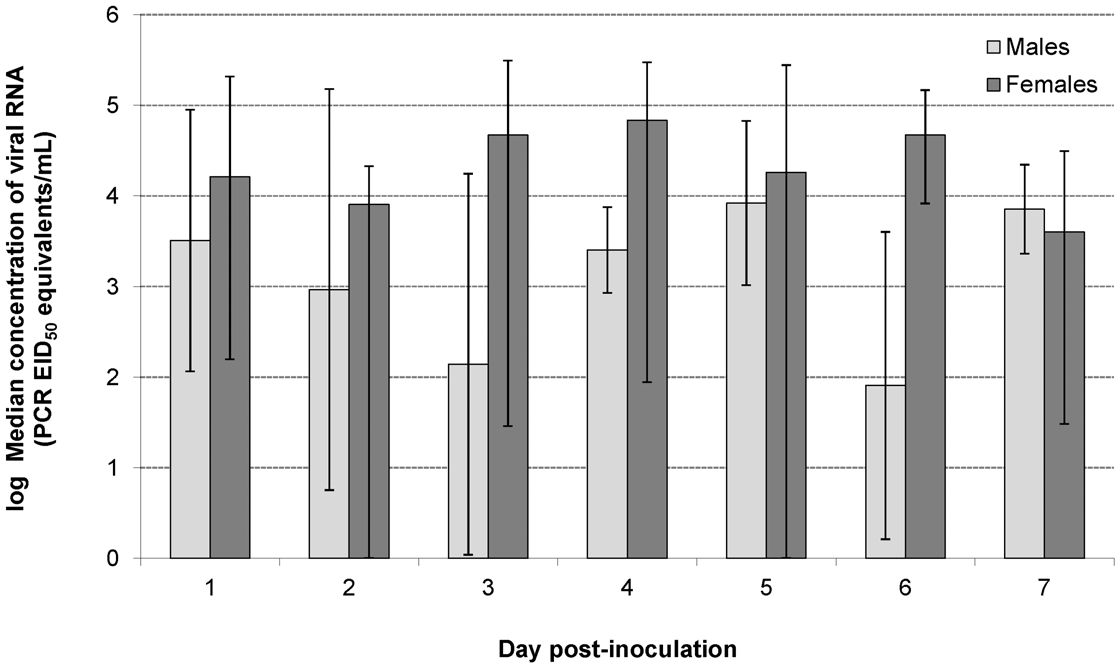
Avian influenza viral replication in lung tissues of male and female mice. Avian influenza viral RNA was significantly higher (p<0.05) in lung tissues of female mice compared to male mice across individuals infected with five subtypes of low pathogenic avian influenza. Data are based on 104 observations from 56 females and 63 males. Error bars are the 1^st^ and 3^rd^ quartiles.

Viral RNA concentrations in lung tissues were significantly higher in female mice compared with male mice across the five LPAI subtypes studied (Figure 2). For females, 88% of individuals had detectable concentrations of viral RNA in their lungs and the median concentration across all days and subtypes was 10^4.24^ EID_50_ equivalents/mL (range = 0–10^6.51^). For males, 75% of individuals had detectable levels of viral RNA in their lungs and the median concentration was 10^3.18^ EID_50_ equivalents/mL (range = 0–10^5.95^). Median daily replication rates were more variable for male mice (range = 10^2.14^–10^3.92^) than for female mice (range = 10^3.60^–10^4.83^).

## Discussion

This study provides evidence that avian influenza viruses may be naturally transmitted to mice at outbreak sites and that wild house mice can efficiently replicate avian influenza viruses without prior adaptation. Even though our sample size was low and we were sampling nearly a month after the outbreak was first detected, the house mice in our mammal survey at the Idaho outbreak site showed a positive response for influenza A antibodies. We recommend that future outbreaks allow for significant rodent sampling on the premises as soon as an outbreak is detected to further our understanding of the role rodents may play in avian influenza dynamics at outbreak sites. Furthermore, low sera sample volumes for the mice prevented us from subtyping the samples, which may have provided stronger support for past avian influenza virus infection. The ELISA used in this study was type-specific for antibodies to influenza A; therefore we cannot rule out that the mice may have been infected with a mammalian influenza strain rather than an avian strain. However, the outbreak farm housed only birds so the probability of the presence of a mammalian influenza strain was likely to be lower than for an avian strain.

Few studies have explored the seroprevalence of avian influenza viruses in synanthropic wildlife at outbreak sites and active areas of infection. Exceptions are surveys by Nettles et al. [55], Henzler et al. [56], and Shortridge et al. [57]. In the first study more than 250 mice and rats from infected farms were sampled after a 1983–84 outbreak of H5N2 virus in domestic poultry in the eastern U.S. No evidence for influenza infection was found in the rodents. However, rodent sampling was conducted 2–3 months post-outbreak, which is relatively late given the monthly survival rate of mice on farms can be as low as 0.55 [58]. Henzler et al. also surveyed mice on poultry farms during an outbreak of H7N2 in poultry in Pennsylvania, U.S. from 1996–1998. Lung and intestinal tissues were tested by virus isolation and all samples were found to be negative. In the Shortridge et al. study, mice and rats associated with poultry markets in Hong Kong were surveyed for exposure to avian influenza viruses after the initial detection of Asian strain H5N1 virus. Again, no virus was isolated from either rodent species, but rats showed evidence of hemagglutination inhibition activity and the ability of both mice and rats to replicate the H5N1 virus was subsequently confirmed [30].

A potential caveat with regard to mammalian sero-surveys is that many assays developed to detect antibodies to avian influenza viruses are optimized for poultry species and may not be validated for other species. Consequently, assay sensitivity and specificity may not be consistent with published parameters. Further, a number of commercial enzyme-linked immunosorbent assays (ELISAs) developed for detection of antibodies to avian influenza viruses use anti-mouse antigens and may not be appropriate for rodents. Hemagluttination inhibition (HI) tests may also be problematic as mouse sera may cause non-specific binding that interferes with HI tests [59]. Only a third of the mice tested in this study were positive for antibodies to avian influenza virus. Further work needs to be done to elucidate whether mice lacking an ELISA response would be protected upon re-exposure to influenza virus.

Rodents could contribute to avian influenza virus transmission on and among farms in a number of ways. Mice and rats could spread virus via mechanical transmission [60]. Alternatively, they could contribute to viral spread by becoming infected via scavenging on infected poultry carcasses [2] or by contact with contaminated water sources [61] and subsequently transmitting virus if they are scavenged or predated. Mice are a very common prey species for a variety of raptors, meso-predators, and other mammalian carnivores and are also commonly eaten by barnyard chickens. The high concentrations of viral RNA detected in the lungs of experimentally infected house mice indicate that wild mice may have the potential to replicate sufficient virus for transmission to other species.

We tested five subtypes of avian influenza virus in order to compare the replication potential of subtypes derived from both wild birds and chickens. Our results indicate that wild mice were able to more efficiently replicate viruses originating from wild birds than from poultry. Viral RNA was detected in 94% of lung, 86% of nasal turbinate, and 78% of trachea tissues from mice inoculated with wild bird viruses compared with 51% of lung, 44% of nasal turbinate, and 20% of trachea tissues from mice inoculated with chicken viruses. Given the wide range of replication potential for different avian influenza virus subtypes and different strains within subtypes, this pattern of higher viral replication of wild bird viruses compared with poultry viruses may be due to chance. Further study is needed to confirm this general result, but if the pattern holds, it may indicate that mice are more likely to introduce an avian influenza strain from wild birds to poultry than to spread poultry viruses among and between farms.

Studying wild house mice rather than laboratory mice is essential to understanding the potential role that mice may play in avian influenza virus outbreak dynamics because wild house mice and laboratory mice may exhibit differential immune function [42]. Standard laboratory mice, including BALB/c mice, have a defective Mx1 gene (Mx1^−^) that reduces their ability to resist influenza virus infections [62], [63], [64], [65]. On the other hand, wild mice with an intact Mx1 gene (Mx1+) are resistant to influenza virus infection [63], [64]. Consequently, the results of studies based on experimental infections of laboratory mice may not accurately reflect viral replication in wild mice. In addition to differing genetics, natural populations of wild mice are likely to include a broad spectrum of heterogeneities that might influence viral replication. For example, sex, age, reproductive status, behavior, nutritional condition, disease status, and parasite load are all likely to affect immune function [42], [43], [66]. In this study, wild house mice were fed standard laboratory diets for 2–5 weeks prior to testing and were dusted for parasites, so individual differences due to nutritional status or parasite loads were likely diminished. However, we were able to evaluate sex and found that female mice exhibited higher viral replication rates compared to males. While none of the females in this study were pregnant at the time of inoculation, a few individuals gave birth during the quarantine period and a range of hormonal conditions were likely. Previous studies of influenza in mice indicate that morbidity, mortality, and viral loads are higher for pregnant females compared with non-pregnant controls [67], [68]. Differential replication rates may be important to consider in the development of risk assessment and transmission models because individuals with higher viral loads may be more likely to cause an infection post contact with a potential host. If so, the basic reproductive number, or the mean number of secondary infections caused by an infected individual, may be different for males and females and that difference might be important in epidemiologic models.

In summary, the ability of wild mice to efficiently replicate avian influenza viruses without adaptation indicates the potential role of wild house mice in avian influenza virus outbreak dynamics and warrants further investigation. In particular, studies that seek to confirm the presence of avian influenza virus antibodies, detect virus, or examine the ability of mice to transmit virus to other species would shed light on whether or not rodent control is an important strategy for avian influenza virus outbreak control.
