# Correction: Low-Pathogenic Avian Influenza Viruses in Wild House Mice

**DOI:** 10.1371/annotation/85fd8f8e-babc-4440-845f-4137f92eb923

**Published:** 2012-07-09

**Authors:** Susan A. Shriner, Kaci K. VanDalen, Nicole L. Mooers, Jeremy W. Ellis, Heather J. Sullivan, J. Jeffrey Root, Angela M. Pelzel, Alan B. Franklin

The Funding statement was incorrect. The correct Funding section should read: "This study was funded by the United States Department of Agriculture. The manuscript was reviewed for general policy statements committing the USDA to action, but otherwise the findings were independently developed by the authors." 

